# Optimizing key parameters for grinding energy efficiency and modeling of particle size distribution in a stirred ball mill

**DOI:** 10.1038/s41598-025-87229-8

**Published:** 2025-01-27

**Authors:** Abdalla M. Elbendari, Suzan S. Ibrahim

**Affiliations:** Minerals Beneficiation and Agglomeration Department, Minerals Technology Institute, Central Metallurgical Research & Development Institute (CMRDI), P.O. Box 87, Helwan, Cairo, 11722 Egypt

**Keywords:** Sub-micron grinding, Stirred ball mill, Specific energy input, Cumulative oversize distribution prediction, Signature plot, Energy science and technology, Engineering, Materials science

## Abstract

Fine grinding using a stirred ball mill can enhance ore liberation but incurs high energy consumption, which can be minimized by optimizing operating conditions. This study explores the impact of key operational parameters—grinding time, stirrer tip speed, solid concentration, and feed size—on grinding efficiency, evaluated using specific energy inputs, in stirred milling of Egyptian copper ore. The particle size distribution (PSD) of ground products was simulated using the Gates–Gaudin-Schuhmann model (GGS) and the Rosin-Rammler-Benne (RRB) function. Taking minimum energy consumption into account, the finest particles (100% ~1 μm) were achieved at the maximum stirrer speed of 500 rpm and a moderate solid concentration of 33.3% after 17 h of grinding, consuming approximately 1225 kWh/t. Experimental data demonstrated a linear correlation between the natural logarithm of the cumulative retained fraction and particle size (µm). The proposed model accurately describes PSDs across different solid concentrations and grinding durations.

## Introduction

The depletion of high-grade ores has necessitated the use of low-grade ores. These low-grade ores have small liberation sizes, and thus require fine grinding, which is an energy-intensive process^1–3^. Conventional comminution equipment has limitations on the achievable fine grinding size and consumes a large amount of energy for this purpose. In a mining operation, 50–60% of the energy is consumed by the comminution process alone^4,5^.

The particle strength increases as particle size decreases^6^. This is because smaller particles have fewer microcracks^7^. Consequently, fine grinding becomes a more complex process for improving material liberation. Ball mills are inefficient for fine grinding, showing a notable drop in energy efficiency when producing particle sizes smaller than 30 μm^8^, making them less economically viable for effective liberation.

Stirred media mills are extensively used in the mining industry for fine and ultrafine grinding to enhance mineral liberation and achieve particle sizes of 10 μm or smaller^8,9^. These mills generally consume 30–40% less energy than ball mills when producing the same product size in fine grinding processes^10^.

Stirred mills achieve energy savings by using of smaller media and high-velocity stirring, which contrasts with the cascading and cataracting movements found in conventional tumbling mills^2^. These mills feature an agitator that rotates rapidly, stirring and circulating the media within the mill. The grinding occurs through rubbing and impingement between the media balls, the media balls and the stirrer, and sometimes between the media balls and the chamber walls, primarily resulting in attrition and abrasion-based breakage^11^. The performance in stirred mills is influenced by factors such as the media size and density, stirrer speed, grinding time, feed size, and media characteristics to achieve the desired fineness with minimal energy consumption^12–14^. Unlike ball mills, in which impact breakage is predominant, stirred mills rely more on attrition^15^. Optimizing the impeller speed is a crucial factor for enhancing the grinding efficiency and performance. Below the optimum stirrer speed, the stress intensities of the grinding media were too low to cause breakage, whereas above the optimum speed, excess stressing led to energy wastage^16^. Jankovic (2003) found that increasing the speed in a high-speed Netzsch mill enhanced the grinding efficiency when milling limestone. Higher impeller speeds lead to more frequent collisions between balls, which likely increases the number of collisions^8^. Additionally, the kinetic energy of the balls increased with stirring speed^17,18^. De Oliveira et al. (2021) observed that increasing the stirrer tip speed significantly enhanced breakage rates across the entire range of particle sizes studied^6^. However, in tests with a pilot tower mill, higher stirrer speeds reduced energy efficiency. This decrease in energy efficiency with increased impeller speed was also observed by Chaponda (2011) for the IsaMill operated between 1500 and 2100 rpm^18^. This observation was supported by^19^.

The solid concentration refers to the mass fraction of solids in the slurry. For breakage during grinding, particles must be trapped between the grinding media and subjected to sufficient stress^20^. In summary, increasing the solid mass fraction affects particles in two main ways. First, it enhances the likelihood that particles will be trapped and stressed between the grinding media, thereby leading to better particle size reduction. Second, a higher solid mass fraction increases the viscosity of the suspension, which can lead to agglomeration. Ding et al. (2007) noted that at low solid mass fractions, the distance between particles is sufficiently large to allow them to move freely, which improves the fluidity of the suspension^21^. However, at high solid mass fractions, the mean interparticle distance decreases, leading to more extensive particle-particle interactions and disrupting the particle free movement. Several researchers^8,21,22^ have noted viscosity issues occurring above a certain critical solid concentration. However, the specific concentration at which these viscosity problems arise varies depending on the milled material. Numerous studies have explored the impact of operational conditions on the energy consumption and particle size distributions in laboratory stirred mills^23–26^. Patino et al. (2022) optimized the ultra-fine grinding of coal using a laboratory-scale stirred media mill (Union Process, Akron, Ohio, 9.5 L). Starting with a feed size of -24.4 μm, they achieved a product size of P_80_ 5.9 μm with an energy consumption of 309 kWh/t. The optimal conditions included a solid concentration of 30%, a stirrer speed of 340 rpm, and a grinding time of 64 min^14^.

Santosh et al. (2020, 2023) investigated the grinding of chromite ore using a 5.3 L NETZSCH vertical stirred mill. Their results showed that operating the mill at a solid concentration of 50.1% and a stirrer speed of 621.5 rpm yielded a product with a particle size of 11.6 μm and an energy consumption of 21.8 kWh/t. The study concluded that reducing solid concentration and stirrer speed enhanced energy efficiency^2,11^. Hasan et al. (2017) observed that a higher tip speed (3 m/s) and a lower solids concentration (50%) produced finer limestone particles less than 100 μm at 10.8 kWh/t in a batch vertical stirred mill^27^.

Yang et al. (2017) reported achieving a particle size of approximately 1.8 μm from a feed with a d_80_ = 6.8 μm for calcium carbonate with an energy consumption of 300 kWh/t using a lab-scale mill (IMERYS, 2.9 L). The process was conducted under conditions of lower solid concentrations (65%) and a lower stirrer tip speed of 5.23 m/s^28^.

Katircioglu-Bayel (2020) investigated calcite grinding in a laboratory batch mill, achieving a P_50_ of 0.3 μm with a high energy input of 1340 kWh/t, under conditions of 25% solid concentration and a stirrer speed of 600 rpm. The study highlighted the potential for achieving a 22% energy savings through parameter optimization, including adjustments to stirrer speed and grinding time^29^.

Celep et al. (2011) used a Box-Behnken design to evaluate the effects of stirrer speed, ball charge ratio, and grinding time on particle size reduction for refractory Au/Ag ores, achieving a d_80_ of 3.37 μm at a stirrer speed of 745 rpm, a ball charge ratio of 80%, and a grinding time of 10.5 min^30^. Similarly, Choi et al. (2007) demonstrated enhanced energy efficiency during calcite powder grinding, starting with a feed size of d_50_ = 10.82 μm and achieving a product size of d_50_ = 350 nm at 700 rpm, 15% solids, and a grinding time of 480 min^31^.

Zheng et al. (1996) established correlations between power input and grinding parameters for calcite, showing that higher solid concentrations initially increased surface area but declined beyond 75%^19^. Lastly, Guo et al. (2024) found that higher solid concentrations enhanced particle breakage in quartz grinding, aligning well with predictions from the Rosin–Rammler–Benne model^26^. These contradictory observations suggest that there may be an optimal stirrer speed and solid concentration for achieving maximum energy efficiency. Therefore, this study aims to optimize key parameters influencing grinding performance in a stirred ball mill, including impeller tip speed, solids concentration, and feed size. The study involves assessing energy consumption and particle size reduction to enhance energy efficiency, as well as developing a predictive model for the particle size distribution of the ground products.

## Materials and methods

In this study, the mill feed sample was copper ore sourced from the Abu-Swayel deposit, situated in the southeastern region of Egypt’s Eastern Desert. This deposit is located approximately 185 km south of Aswan, near Wadi Haimour^32^. The ore is characterized by a density of 2.9 g/cm³ and a Work Index of approximately 16.7 kWh/t.

**Fig. 1:**
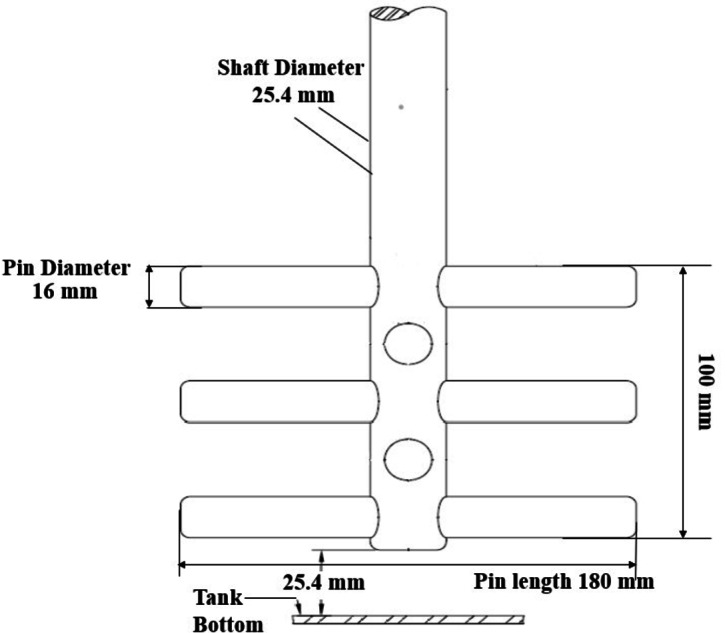
Diagram of the mill impeller used in the study.

The grinding tests were carried out using a laboratory-scale Stirred mill from Union Process, Akron, Ohio, which is commonly referred to as an Attritor. This versatile mill is capable of producing consistent results across different batches and is suitable for both wet and dry grinding under controlled operating conditions. The mill has a 2.5-gallon (9.5 L) tank with a working capacity of 1.1 gallons (4.2 L). Figure 1 illustrates the diagram of the mill impeller, featuring a stainless-steel shaft equipped with five adjustable stainless-steel arms. These arms can be modified to accommodate different grinding media sizes. The electronic variable speed drive system allows for precise control of the shaft speed in the RPM. Additionally, the mill has a bottom discharge grid for sampling and discharge, water-cooled jackets for temperature control, and an LCD that displays the impeller speed (rpm), motor frequency (Hz), and power draw in kilowatts (kW).

The performance of stirred media mills is governed by a variety of parameters that interact in complex ways, as highlighted in the literature^8,33^. Molls and Hornle (1972)^34^, as cited by Jankovic (2003), identified up to 44 factors influencing stirred milling performance^8^. However, research has primarily focused on key parameters considered most critical for optimizing grinding efficiency. These include media size, stirrer speed, slurry density, and the rheological properties of the slurry. Among these, stirrer speed is consistently recognized as one of the most significant variables^8,33,35,36^, as it directly impacts the breakage mechanism and energy distribution within the grinding chamber^37^.

In this study, stirrer speed and solid concentration were selected as key parameters based on insights from these foundational works. The literature underscores their critical roles in influencing grinding efficiency and product quality, aligning with the established understanding of the factors that most significantly affect stirred media mill performance.

The batch grinding was conducted under wet conditions using 3 mm diameter alumina balls. The experiments explored various parameters, including the grinding time (hour), impeller speed (rpm), slurry solid mass fraction (% solid in slurry), and feed size (µm). The mill’s specifications and the experimental conditions are detailed in Table 1.

**Table 1:** Wet attritor ball mill grinding test conditions.

Item	Description	
Mill	Inner diameter (D), mm	240
	Length, mm	210
	Tank capacity, L	9.5
	Working capacity, L	4.2
	Model	1-S
	Variable speed drive system, rpm	Up to 550
Media (Balls)	Material	alumina 99.5% purity
	Specific gravity, g/cm^3^	3.68
	Mass of balls, Kg	6
	Assumed porosity	40%
	Ball size, mm	3
	Hardness, Hv	1350 Kg/mm^2^
	Ball filling volume fraction*(J)*, [%]	35
Material	copper ore sample	
	Specific gravity, g/cm^3^	2.9
	Initial feed size	−500 + 75 μm, −75 μm
	% solid in slurry	20, 33.3, 50%
	Slurry density, g/cm^3^	1.15, 1.28, 1.48

The grinding media (ball) filling volume fraction (*J*), calculated using Eq. (1), indicates the proportion of the volume occupied by the media, with a bed porosity of 0.4^38,39^.

1 $$\:J=\:\frac{Mass\:of\:media\:\left(gr\right)/\:Density\:(gr/{cm}^{3})}{Mill\:volume\:\left({cm}^{3}\right)}\times\:\frac{1}{0.6}$$

The specific energy consumption (E_S_) during the wet grinding process was measured to assess the grinding efficiency. It was calculated using power input readings from the tests and the unloaded power input, which was recorded under “free-load” conditions. This no-load power draw, representing the energy needed to operate the shaft, must be subtracted to determine the specific energy required for reducing the particle size^40^. Equation (2) was used to calculate the specific energy:

2 $$\:{E}_{s}=\:\frac{E-{E}_{0}}{{m}_{p}}(kWh/t)$$

where *m*_p_ is the product mass (mass of solid in the pulp), E is the energy used at the time t, and E_0_ is the no-load energy.

The stress intensity (SI) was calculated using Eq. (3) developed by Kwade et al. (1997), which mathematically relates media size, media density, and rotational rate to stress intensity^41^.

3 $$\:S{I}_{m}={{D}_{m}}^{3}\left({\rho\:}_{m}-p\right){{v}_{t}}^{2}$$

SI_m_ Grinding media stress intensity (Nm), D_m_ Grinding media size (m), υ_t_ Impeller tip speed (m/s), *Ρ* Density of the slurry (Kg/m^3^), *ρ*_*m*_ Density of grinding media (Kg/m^3^).

The particle size distribution was analyzed using different models to identify the best-fitting model for the fine product size after grinding. In some cases where the distribution is skewed, a linear plot can be obtained by plotting the log of the cumulative undersize against the log of the screen aperture. Such plots are known as Gaudin–Schuhmann plots. In most cases this would yield a straight line. The second model is Rosin–Rammler plots (RRB) or Weibull is an alternative plot, especially suitable for finely ground particles, is to plot log [log 100/ (100 − *y*)], or log [log 100/*R*], against the log of sieve size, where *y* is the cumulative % passing and *R* is the cumulative % retained^25^.

The RRB distribution is expressed by Eq. (4):

4 $$\:R=100\text{exp}\left(-{\left(\frac{x}{{x}_{i}}\right)}^{k}\right)\:$$

where *R* is the cumulative sieve retained of particles, %. *x* is the particle size, µm. *x*_*i*_ represents the particle size in correspondence to 36.78% retained weight, *k* is the distribution index.

The Mastersizer 2000 particle size analyzer was employed to analyze the particle sizes of both the copper feed and the ground products. Before measurement, samples were thoroughly dispersed using ultrasound unit for 2 min in 500 ml of water to break up the aggregates. Each test was repeated three times, and the reported values were the mean averages. The Mastersizer 2000 can measure particle sizes ranging from 0.1 to 1036 μm.

The mineral composition of the sample was identified using X-ray diffraction (XRD). The analysis involved examining the X-ray diffraction patterns of the specimens using ASTM powder pattern data. Both bulk mineral samples and pure mineral phases were finely ground, mounted randomly on an aluminum holder, and analyzed. The X-ray diffractometer (Bruker AXS D8 Advance, Germany) with Cu Kα radiation (λ = 1.5406 Å) and a secondary monochromator was used to identify different phases in the samples within the 2θ range of 2 to 75°.

## Results and discussion

The efficiency of particle breakage in grinding processes is strongly influenced by key parameters such as stirrer speed and solid concentration (slurry density). Higher stirrer speeds increase kinetic energy, enhancing impact forces and promoting finer particle sizes, but also lead to higher energy consumption, requiring careful optimization for balance. At higher speeds, fracture mechanisms become more significant, while attrition dominates at lower speeds^15^. Furthermore, stirrer speed affects the distribution of the grinding zone, influencing milling performance^42^.

Similarly, solid concentration impacts the slurry rheology, energy transfer efficiency, and particle dynamics within the grinding chamber. As lower concentrations typically promote higher breakage rates due to improved particle-media interactions. Conversely, higher solid concentrations increase slurry viscosity, reducing effective collisions and grinding efficiency, potentially hindering effective breakage if shear stress does not reach the critical breakage stress of certain materials^43^. A balanced slurry density is essential for optimizing energy utilization and achieving the desired particle size distribution. Studies have shown that optimal solid concentration and tip speed vary depending on the material being processed. For instance, research on iron ore and barium titanate identified specific conditions that maximize grinding efficiency^43,44^. Tailoring these parameters to account for material characteristics such as hardness and initial particle size is crucial for achieving optimal performance while minimizing energy consumption and wear on mill components.

### Effect of impeller tip speed

The impeller speed is one of the key factors that affect the grinding performance^45,46^. It directly affects the amount of energy consumed during the process and the maximum velocity that the grinding media can reach, thereby increasing the momentum of the grinding media.

In this study, a grinding media diameter of 3 mm, a ball filling volume fraction of 30%, and a solid density of 1.48% (50% solids) were used to investigate the impact of stirrer tip speed. The influence of impeller speed was assessed by operating the mill at three distinct levels: a low speed of 2.54 m/s (270 rpm), a standard tip speed of 3.62 m/s (385 rpm), and a high speed of 4.71 m/s (500 rpm).

**Fig. 2:**
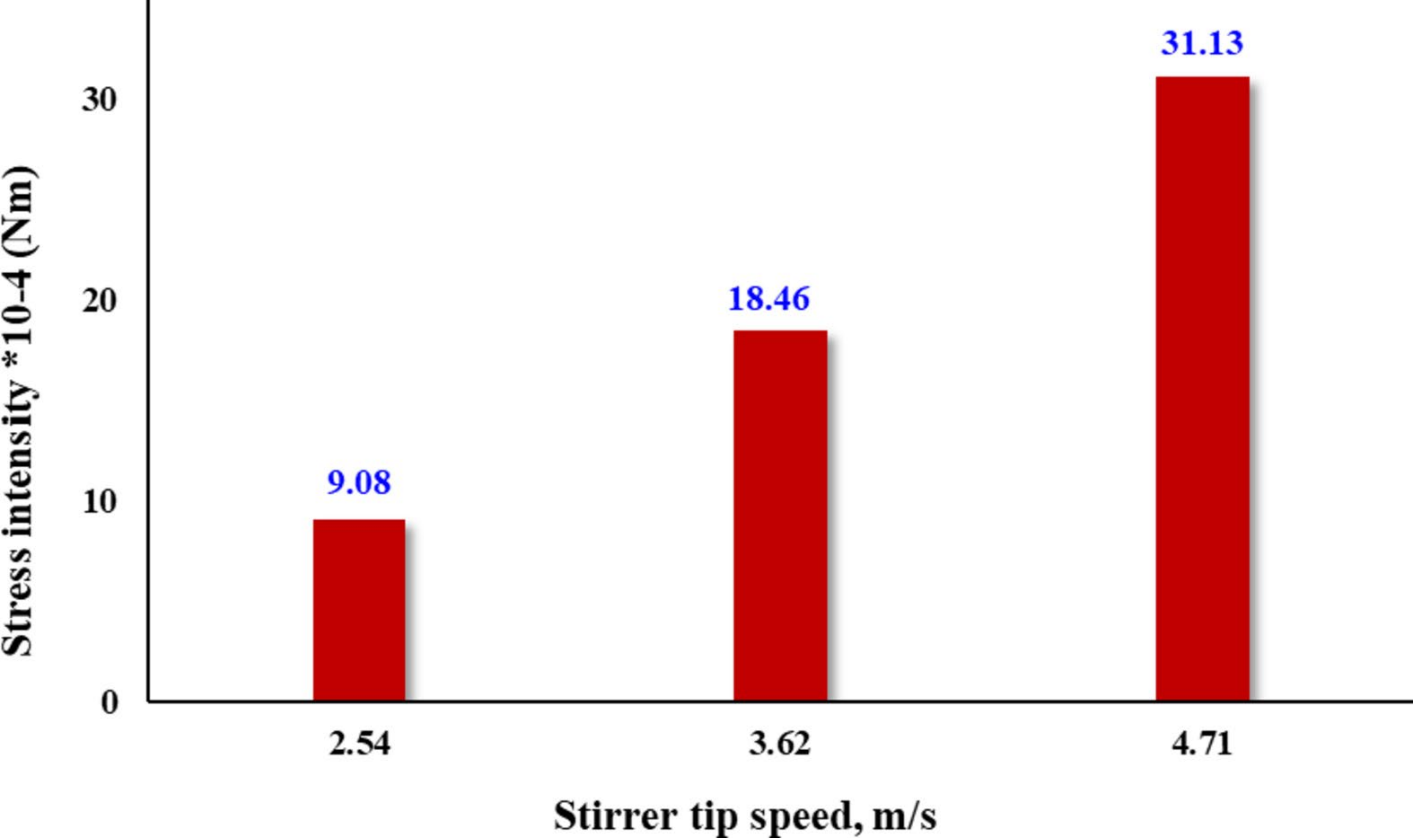
Stress intensity at different stirrer tip speed.

**Fig. 3:**
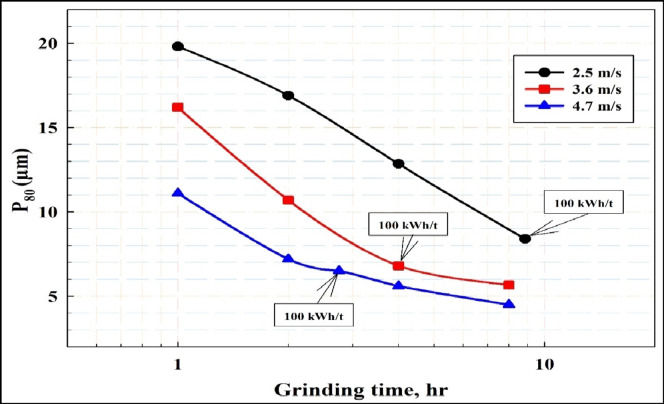
The impact of stirrer speed on grinding performance at various times.

**Fig. 4:**
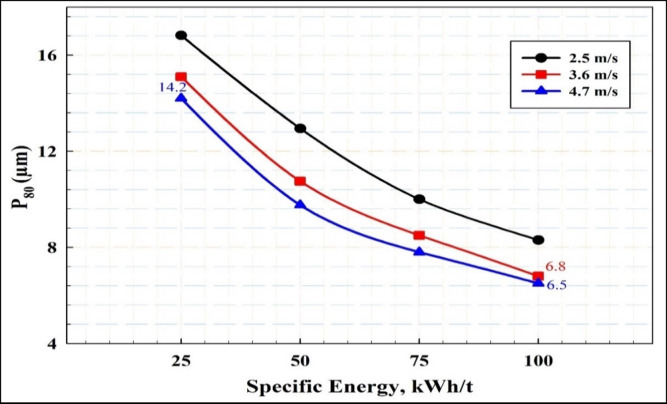
The effect of the specific energy consumption on grinding performance at different impeller speeds.

Figure 2 displays the calculated stress intensity at various stirrer speeds. It is evident that increasing the impeller tip speed results in a roughly threefold increase in stress intensity, rising from 9 × 10⁻⁴ Nm at a low tip speed of 2.54 m/s to 31.13 × 10⁻⁴ Nm at a higher tip speed of 4.71 m/s. Figure 3 illustrates the impact of stirrer speed on grinding performance at various times. It has been shown that at all studied times the particle size decreased with increasing stirrer speed. Figure 4 depicts the effect of stirrer speed on grinding efficiency at different energy inputs. The results indicate that decreasing the impeller speed leads to an increase in specific energy throughout the tested speed range. Finer particle sizes were achieved with lower energy input at higher impeller speeds. Specific energy consumption decreases as the impeller speed increases from 2.54 to 4.71 m/s, with a notable drop in specific energy consumption occurring between 2.54 and 3.62 m/s. At a specific energy input of 100 kWh/t, the produced particle size d_80_ is 8.4 μm at a low impeller tip speed, 6.8 μm at a standard tip speed, and 6.5 μm at a high tip speed. The corresponding processing times are 9 h, 4 h, and 2 h 45 min, respectively, as shown in Figs. 3 and 4. (Gao and Forssberg, 1993) observed that increasing the tip speed of a stirred ball mill results in slightly lower energy efficiency for particle breakage^35^. This is due to greater energy consumption from collisions between the grinding media and heat generation to overcome mechanical challenges, rather than enhancing the particle breakage rate. Nevertheless, higher grinding speeds significantly boost the grinding rate, making them advantageous for operation. It could be suggested that grinding rate and efficiency (energy utilization) decrease at low tip speeds, while efficiency improves at moderate and high tip speeds. These results align with (Hasan et al., 2017) which demonstrated that higher mill tip speeds enhanced particle breakage in the stirred ball mill^27^. (Yang et al. 2006; Jayasundara et al. 2010; Guo et al. 2021) concluded that increasing agitator tip speed increased particle velocity, which in turn increased impact energies, compressive forces and power draw^33,47,48^.

### Effect of solid concentration in slurry

In this study, various tests were conducted to assess the impact of solid concentration on grinding performance. The rotation rate of the impeller was set to its maximum of 500 rpm to achieve the fastest and most intense grinding conditions. This equates to a tip speed of 4.71 m/s. The solid concentration was adjusted by changing the mass of ore while maintaining a constant water volume of 1 L. The impact of solid concentration on milling performance was investigated at three different levels: 20%, 33.3%, and 50%, corresponding to slurry densities of 1.15, 1.28, and 1.48, respectively.

Table 2 displays the calculated stress intensity at different solid concentrations. It is clear that reducing the % solids in the slurry leads to an increase in stress intensity, rising from 31.13 × 10⁻⁴ Nm at a high solid concentration of 50% to 35.92 × 10⁻⁴ Nm at a low solid concentration of 20%.

**Fig. 5:**
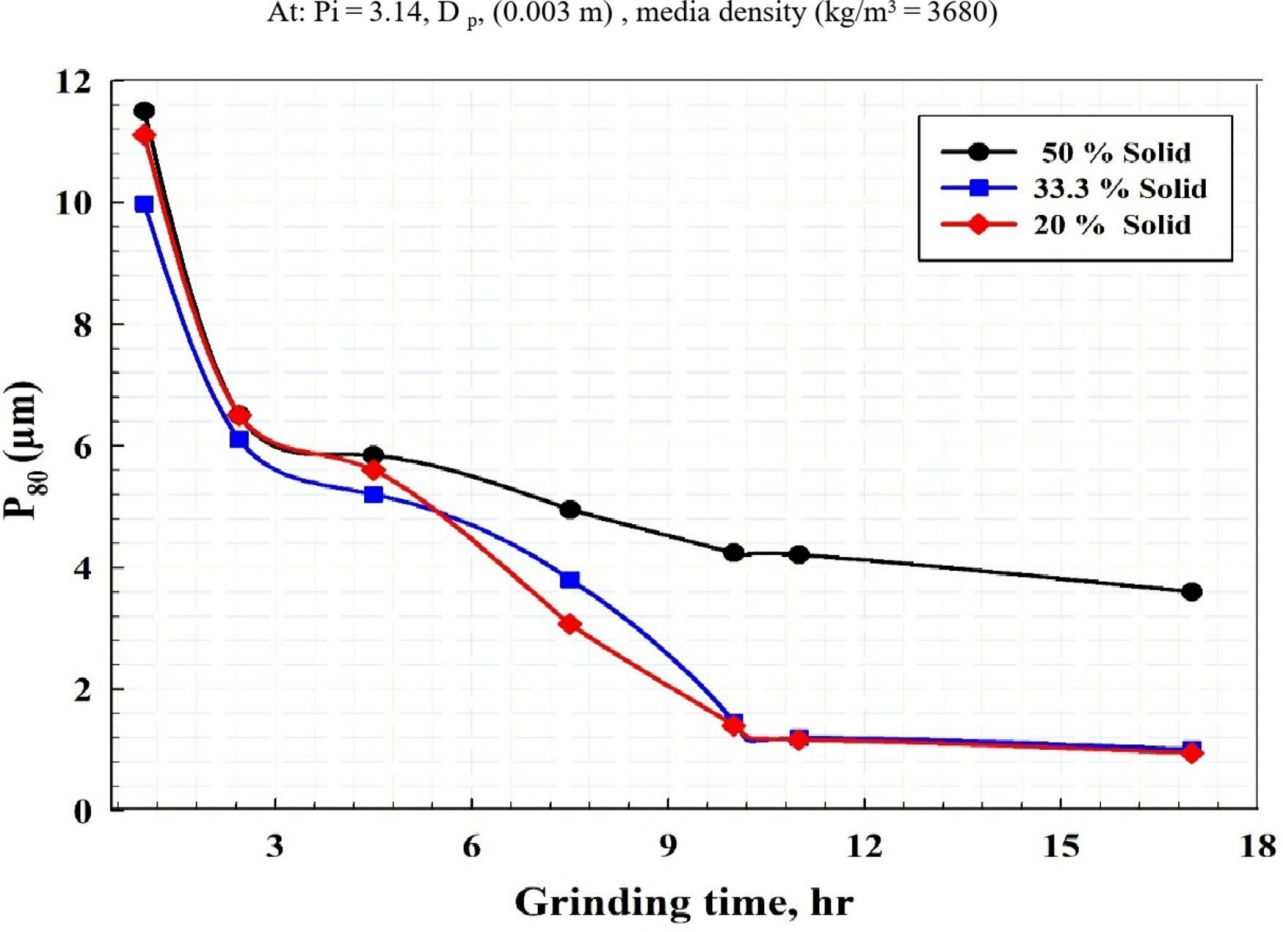
Effect of solid concentration on product size (P_80_) at various grinding times.

Figure 5 illustrates the impact of reducing solid content on the product size P_80_ at various grinding times. By decreasing the % solid, the particle size drastically decreased. However, the increase in particle size with higher solid concentration due to the increase in viscosity of the slurry.

**Fig. 6:**
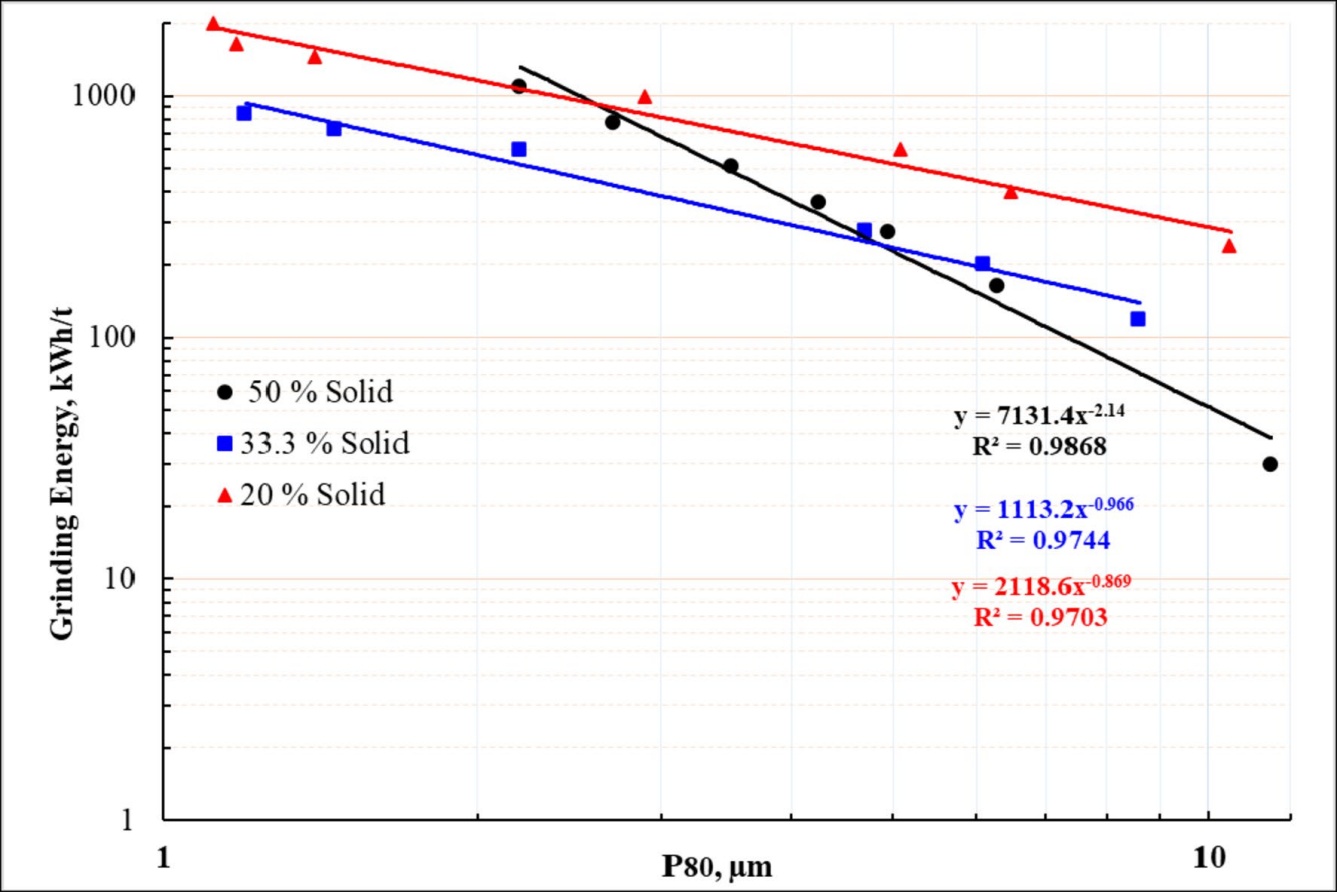
Signature plot (Specific energy in relation to grind size, µm for batch attritor ball mill at different solid concentration).

Figure 6 presents the results as net specific energy versus grinding size, characterized by P_80_, at various slurry solids percentages. This type of graph, referred to as a “signature plot”, is considered a widely accepted and proven method for assessing grinding efficiency. This plot is unique to the ore, pulp conditions, and selected media, and it can be used for scaling up stirred mills to an industrial scale^49–51^. Since this data is used for mill sizing, its accuracy is of utmost importance. The plot of particle size (P_80_) on the logarithmic x-axis against net energy on the logarithmic y-axis produces a straight line with high R² values. It has been shown that grinding efficiency improves, meaning less energy input is required, as the slurry % solids increase up to a certain point. However, beyond this point, the efficiency starts to decline. The specific energy input considerably increased by decreasing solid concentration. Overall, increasing the energy input leads to a clear reduction in particle size. Grinding with 20% and 33.3% solids concentration produces a finer product compared to 50% solids concentration. This suggests that achieving finer size (less than 1.8 μm) becomes more challenging with higher slurry solids loading at the same energy input. It is noteworthy that the increase in efficiency from 33.3 to 50% solids was not as high as from 20 to 33.3%. Lowering the % solid (from 33.3 wt., % to 20 wt., %) resulted in approximately doubling energy consumption from 400 kWh/ton to 800 kWh/ton to reach particle size of P_80_ = 5 μm.

In conclusion grinding at moderate solid 33.3% give the minimum particle size with minimum energy consumption. An increase in solids % to moderate level (33.3%) leads to higher grinding efficiency due to a greater number of particles in the mill, which in turn raises the probability of particle breakage. However, if the solids concentration exceeds 33.3%, especially during extended grinding times, the particle population in the mill becomes excessively dense, making it difficult to move the charge. This can result in higher power draw and, consequently, reduced grinding and energy efficiency. Taken into consideration the energy consumption, grinding at high solid concentration is preferred to achieve particle of size P_80_ less than 3.3 μm. Achieving finer particle size require grinding at less solid concentration. (Jankovic 2003) stated that the improved grinding efficiency at higher % solids can be attributed to the reduced power draw resulting from the “buoyancy” effect and the increased number of particles within the mill^8^. This latter effect causes an increase in probability that a particle will be broken.

**Fig. 7:**
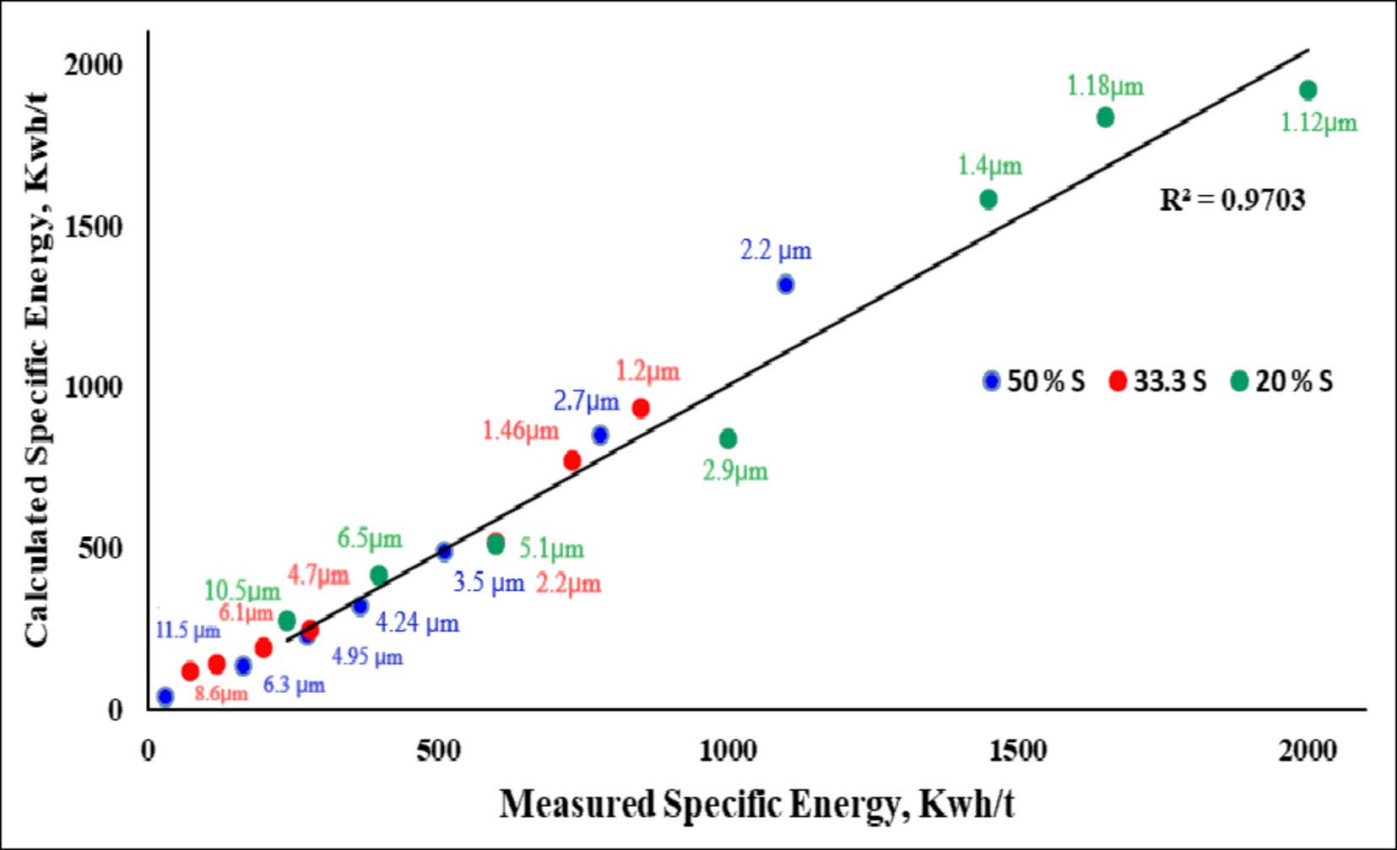
Relationship between measured and calculated specific energy (kWh/t) for achieving specific sizes (µm) at various solid percentages.

**Fig. 8:**
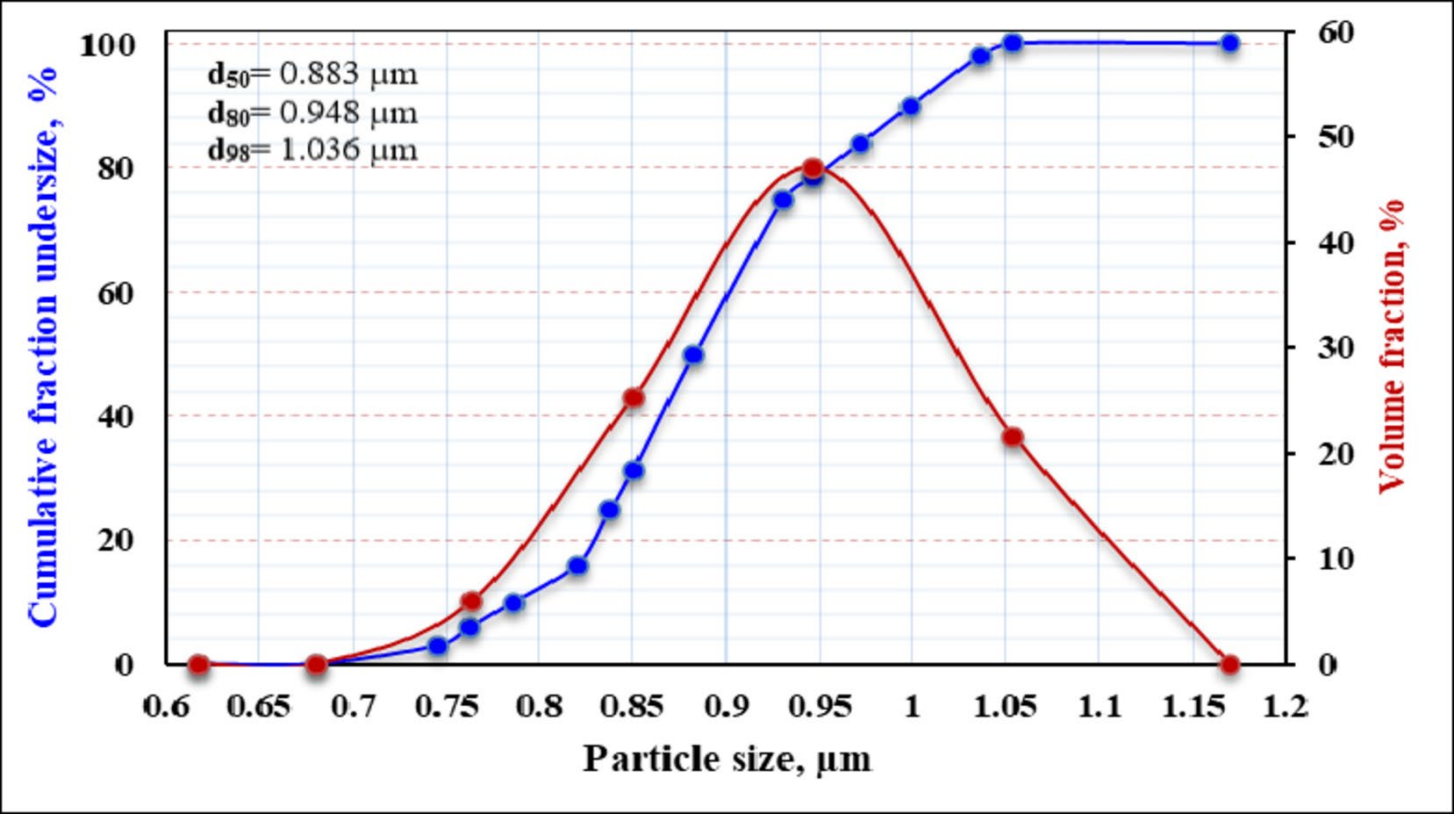
Particle size distribution of the final product.

Figure 7 illustrates the relationship between measured and calculated specific energy (kWh/t) for achieving specific sizes (µm) at various solid percentages. The finest particle size, with 100% of particles ~ 1 μm, was achieved at a solid concentration of 33.3% and a grinding duration of 17 h, beyond which no significant reduction in particle size occurred. The energy consumption was approximately 1225 kWh/t. Figure 8 displays the particle size distribution of the final product, revealing a uniform distribution.

**Table 2:** Stress intensity values at various solid concentrations.

Speed (rpm)	linear velocity (m/s)	Solid (%)	Slurry density (kg/m^3^)	SI* 10^− 4^ (Nm)
500	4.71	50	1487	31.13
500	4.71	33.3	1280	34.07
500	4.71	20	1150	35.92

At: Pi = 3.14, D _p_, (0.003 m), media density (kg/m^3^ = 3680).

### Effect of initial feed size and ore characteristics

**Fig. 9:**
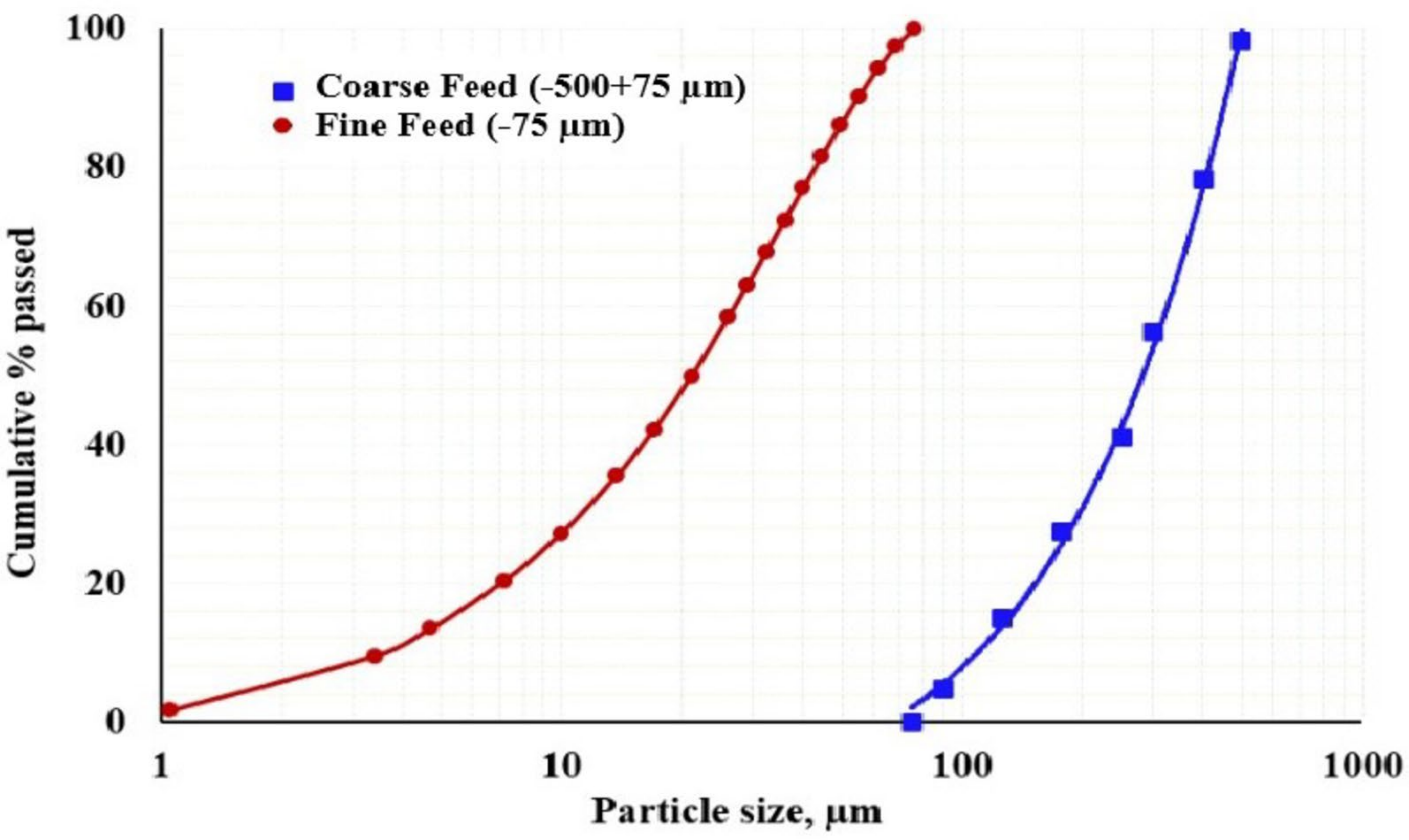
Particle size distribution of the mill feeding ore sample.

**Fig. 10:**
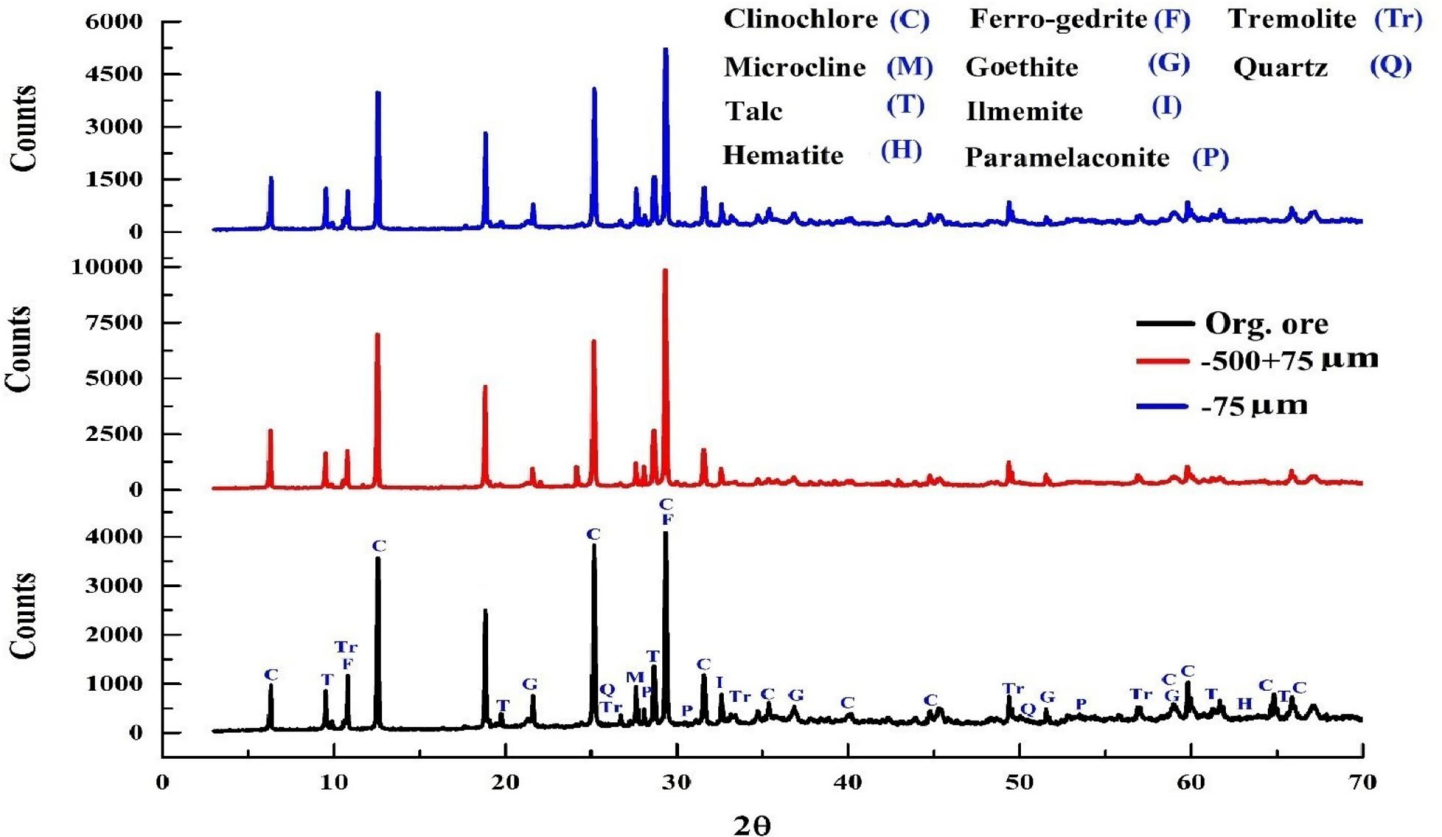
XRD analysis of original copper ore sample, -500 + 74 microns, -75 microns

Grindability is significantly affected by the initial feed size and its properties, such as hardness and composition. Numerous studies, including those by^5,28,52^, have explored how the initial feed size influences the grinding performance of different minerals. The particle size distribution (PSD) analysis of the feed sample, with F_80_ values of 410 μm and 49.3 μm, is shown in Fig. 9. The characteristics and mineralogical composition of the coarse and fine feed samples are detailed in Fig. 10; Table 3.

**Fig. 11:**
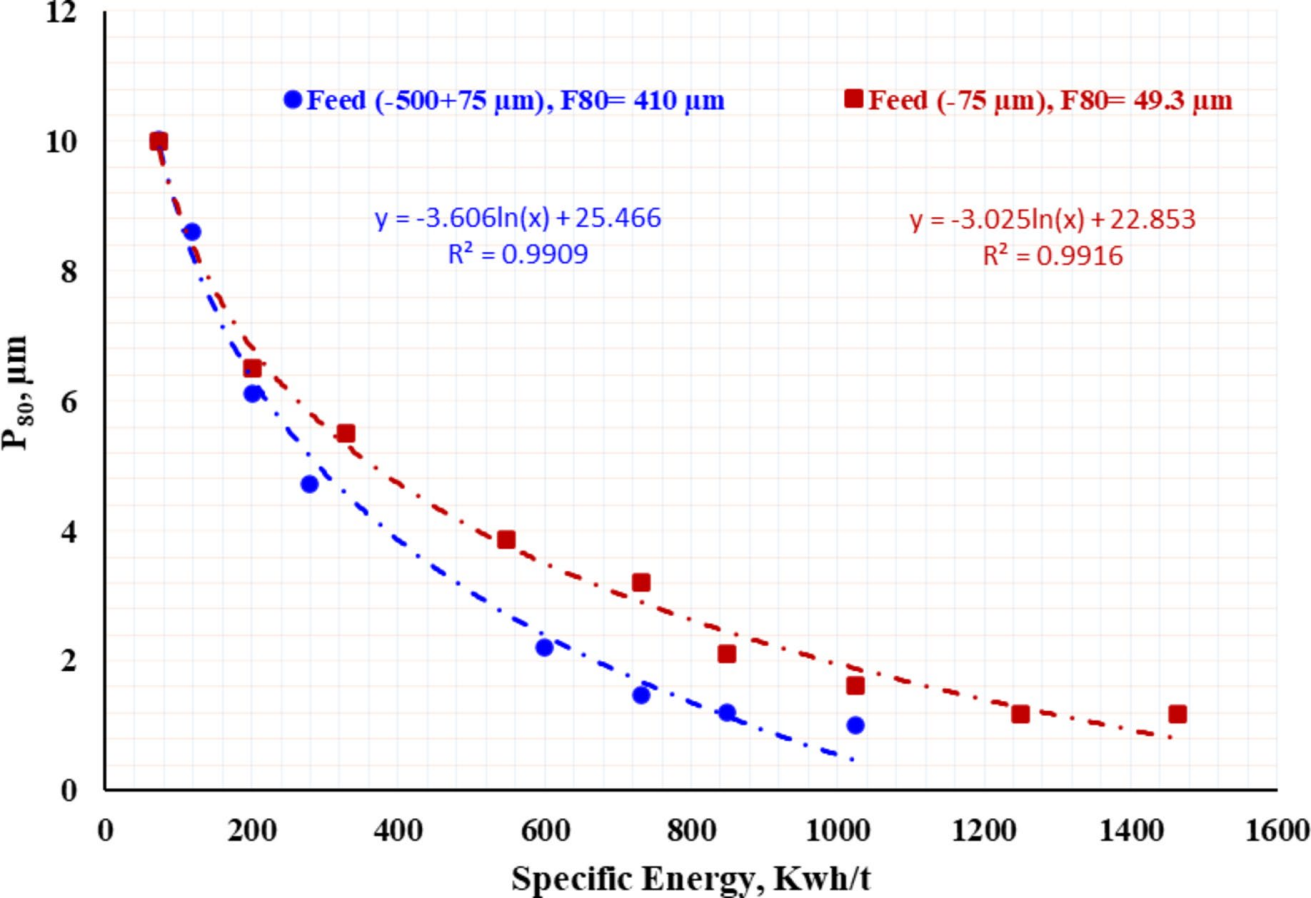
Impact of initial feed particle sizes (− 500 + 75 μm vs. −75 μm) on milling efficiency at 33.3% solids concentration and 4.71 m/s impeller tip speed.

Figure 11 demonstrates how different initial feed particle sizes (− 500 + 75 μm and − 75 μm) affect milling efficiency. The grinding was performed at 33.3% solid concentration and 4.71 m/s impeller tip speed. The results suggest that grinding coarser particles (− 500 + 75 μm) is easier compared to finer particles (− 75 μm). For instance, achieving a particle size of P_80_ ~ 1 μm (with a reduction ratio of F_80_/P_80_ = 410) required around 1025 kWh/t for the coarser feed, whereas the finer feed required 1500 kWh/t, even though the reduction ratio was only 49. This could be attributed to the fact that feeding finer particles, which have a higher specific surface area, leads to a more rapid increase in the pulp’s viscosity compared to coarser feed.

**Table 3:** XRD semi-quantitative analyses and physical characteristics of org, (-500 + 75)- and (-75)-microns products.

Mineral	Formula	Hardness	Org.	-500 + 75 μm	-75 μm
Clinochlore	Mg_6_Si_4_O_10_(OH)_8_	2.0-2.5	29.10	47.70	31.30
Ferro-gedrite	{Fe^2 +^ _2_} Fe^2+^ 3Al_2_} (Al_2_Si_6_O_22_) (OH)_2_	5.5-6.0	27.20	29.80	31.80
Tremolite	Ca_2_(Mg_5_ Fe^2+^) Si_8_O_22_(OH)_2_	5–6	18.30	7.70	15.40
Talc	Mg_3_Si_4_ O_10_ (OH)_2_	1.00	7.90	7.00	7.40
Ilmenite	FeTiO_3_	5–6	7.00	1.60	5.90
Microcline	KAlSi_3_O_8_	6-6.5	5.40	2.60	4.30
Goethite	FeOOH	5-5.5	2.40	1.20	1.80
Quartz	SiO_2_	7	1.60	0.70	1.10
Hematite	Fe_2_O_3_	6.5	1.00	0.70	0.70
Paramelaconite	(Cu^1+^Cu^2+^)_2_O_3_	4.5	0.20	0.30	0.20

### Mathematical model of product size distribution

**Fig. 12:**
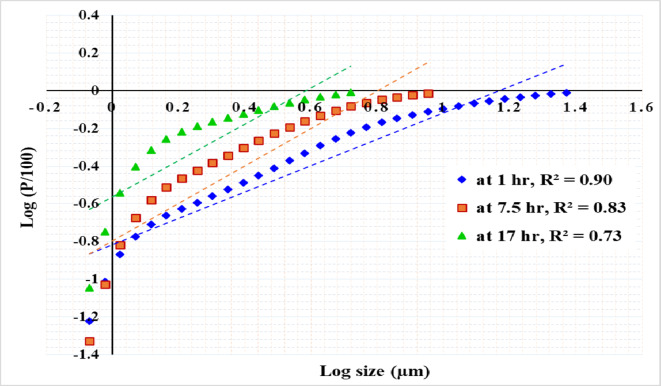
Modeling product size distribution using Gaudin–Schuhman equation (GGS).

Figure 12 shows the results of modeling particle size distribution data using the GGS method. It has been found that for an attritor mill, plotting the logarithm of the cumulative undersize against the logarithm of the screen aperture does not provide an accurate fit for the grinding data curves. This indicates that the GGS method may not be appropriate for simulating all grinding product size distributions.

**Fig. 13:**
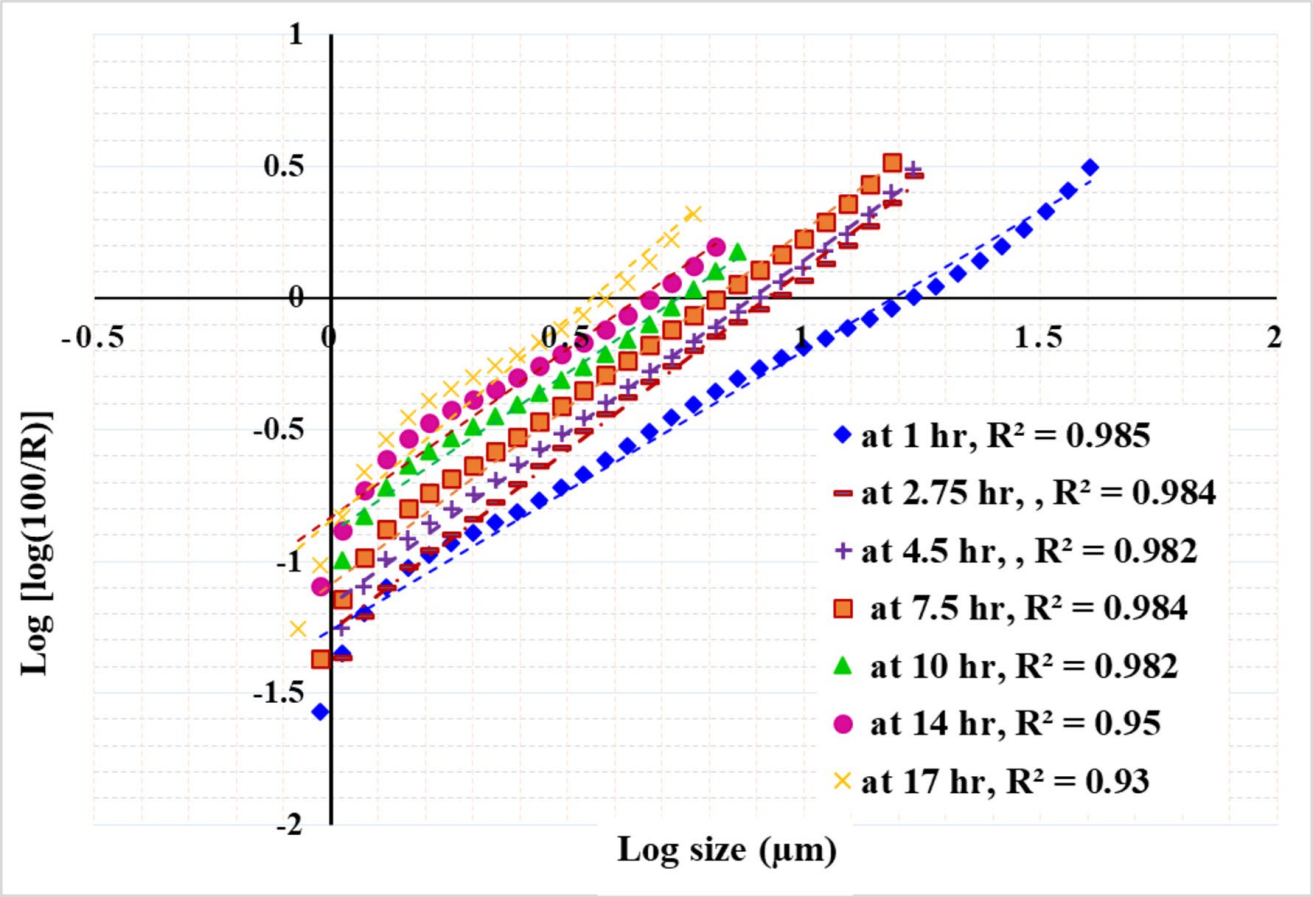
Rosin–Rammler plot for grinding with 50% solid concentration.

**Fig. 14:**
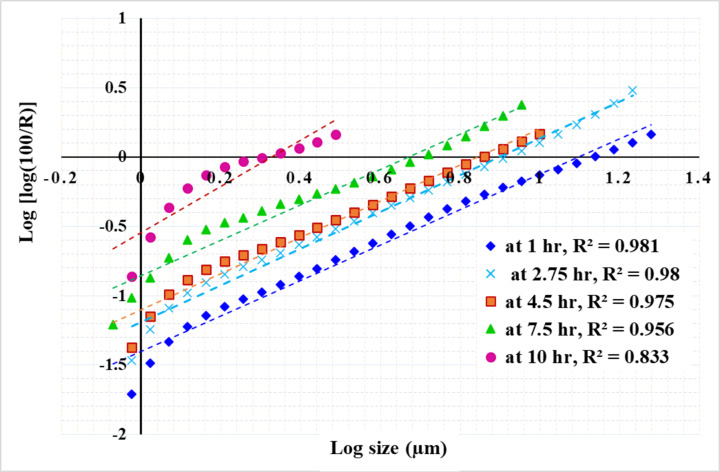
Rosin–Rammler plot for grinding with 33.3% solid concentration.

**Fig. 15:**
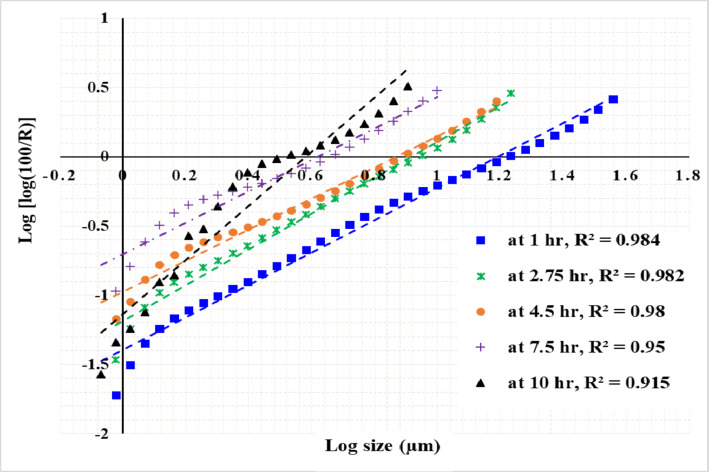
Rosin–Rammler plot for grinding with 20% solid concentration.

Figures 13, 14 and 15 present the results of modeling particle size distribution data using the RRB method. It has been demonstrated that the RRB model provides a good fit for grinding to a product size of d_100_ > 6 μm (d_50_ > 1.4 μm) across all studied pulp densities. However, it does not accurately predict particle sizes below 6 μm, as shown in Figs. 13 and 14. This occurs when grinding exceeds 14 h at a 50% solids concentration or 7.5 h at 33.3% and 20% solids concentrations.

**Fig. 16:**
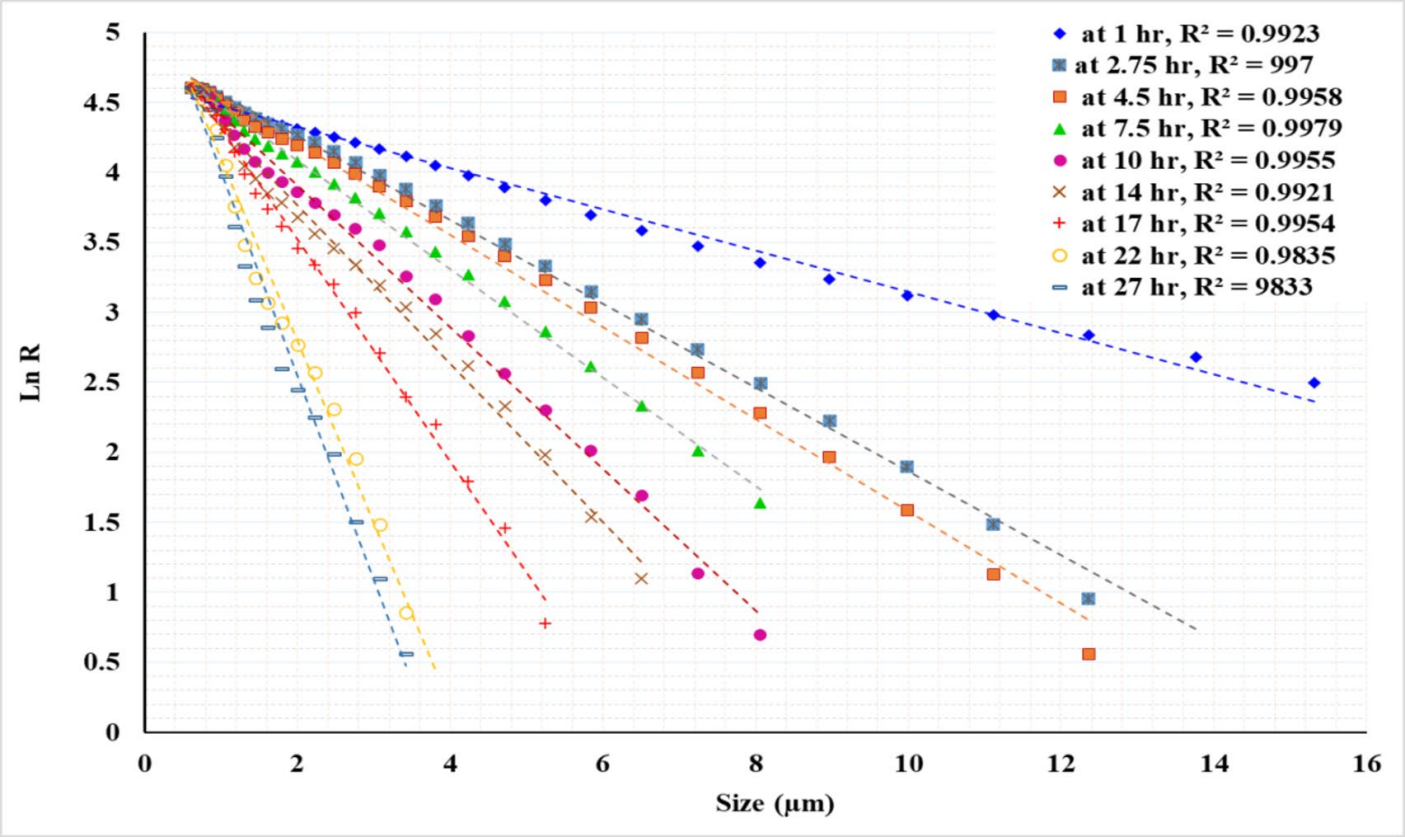
Plot of particle size versus the natural logarithm of cumulative retained (Ln R) at 50% solid concentration for various grinding times.

**Fig. 17:**
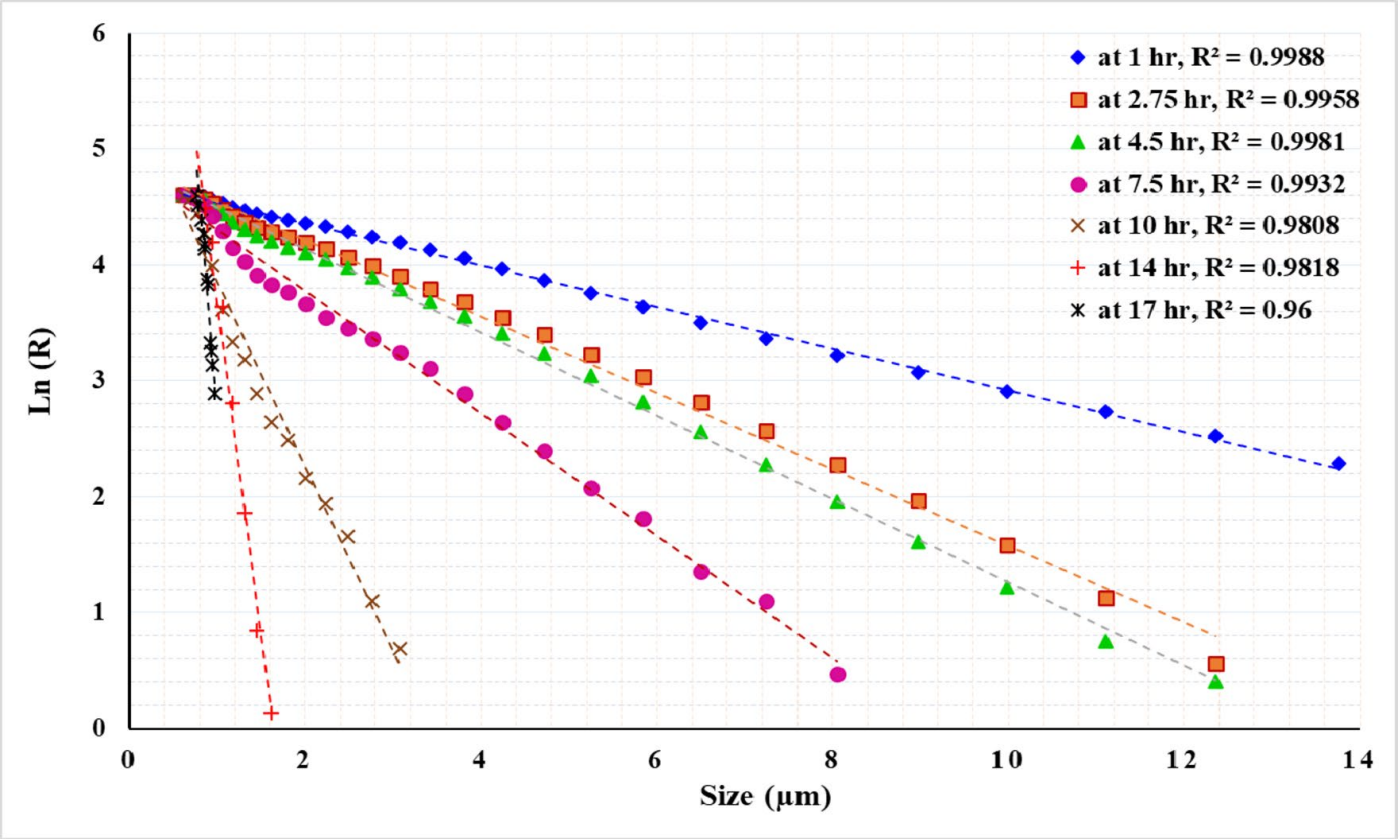
Plot of particle size versus the natural logarithm of cumulative retained (Ln R) at 33.3% solid concentration for various grinding times.

**Fig. 18:**
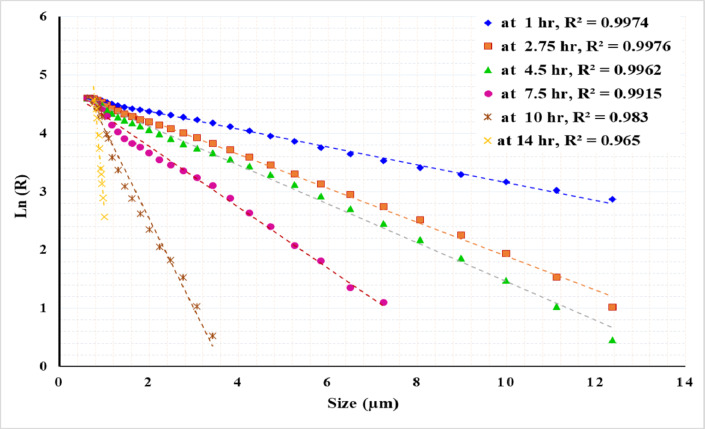
Plot of particle size versus the natural logarithm of cumulative retained (Ln R) at 20% solid concentration for various grinding times.

Figures 16 and 17, and 18 exhibit linear relationships when plotting particle size (in microns) against the natural logarithm of the cumulative retained fraction (Ln R) for solid concentrations of 50%, 33%, and 20%, respectively. The proposed model has been shown to accurately fit all product size distributions across the studied solid concentrations and grinding times. However, the R² value decreased as grinding time increased, resulting in finer particles. The highest R² value of 0.9988 was observed at the shortest grinding time of 1 h, while the lowest R² value of 0.96 was recorded at the longest grinding time of 17 h. The general Eq. (5) can be written as:

5 $$\:R={exp}^{-kX+m}$$

Where: R: the weight% retained, X: particle size, *k*: the slope of the line, representing the rate of change of ln(R) with respect to x, *m*: the intercept of the line, indicating the value of ln(R) when x = 0.

Using a statistical analysis program, the following Eqs. (6) and (7) were derived to predict the values of *k* and *m* for grinding times of up to 7.5 h:

6 $$k\, = \, - \,0.{\text{13}}0{\text{425}}\, + \,0.0{\text{973}}0{\text{3 A}}\, + \,0.0{\text{1341}}0{\text{ B}}\, - \,0.000{\text{597 A}} \cdot {\text{B}}\, - \,0.00{\text{3438A}}^{2} - 0.000{\text{182 B}}^{2}$$

7 $$m\, = \,{\text{4}}.{\text{4844}}0\, + \,0.0{\text{68289 A}}\, + \,0.0{\text{1}}0{\text{261 B}}\, + \,0.000{\text{6}}0{\text{2 A}} \cdot {\text{B}}\, - \,0.00{\text{7718 A}}^{2} - 0.000{\text{176 B}}^{2}$$

For grinding times exceeding 7.5 h, the Eqs. (8) and (9) for predicting the values of *k* and *m* are as follows:

8 $$k\, = \, - \,{\text{3}}0.{\text{15647}}\, + \,{\text{2}}.{\text{9626}}0{\text{ A}}\, + \,0.{\text{731419 B}}\, - \,0.0{\text{58752 A}} \cdot {\text{B}}\, + \,0.00{\text{1}}00{\text{1 A}}^{2} - 0.00{\text{2519 B}}^{2}$$

9 $$m\, = \, - \,{\text{2}}0.0{\text{4731}}\, + \,{\text{2}}.{\text{5}}0{\text{269 A}}\, + \,0.{\text{542267 B}}\, - \,0.0{\text{49276 A}} \cdot {\text{B}}\, - \,0.0000{\text{12 A}}^{2} - 0.00{\text{1}}0{\text{4}}0{\text{ B}}^{2}$$

Where: *A* : the grinding time (hours), *B*: the solid concentration (%), ranging from 20 to 50%.

Table 4 presents the actual and predicted values of *k* and *m*. By applying these values in Eq. (4), the percentage retained at various times can be predicted. For instance, the provided data in the table includes predictions for d_50_, d_80_, and d_90_ at different times and solid concentrations. The results show a good fit between the actual and predicted values, demonstrating the reliability of the developed equations for modeling the grinding process. It can be concluded that the proposed model accurately predicted the product size distribution generated by stirred milling, including particles down to 1 micron. Table 5 presents a summary of various studies conducted on different ores, emphasizing the required solid concentrations, stirrer speeds, grinding times, and energy consumption needed to achieve a specific product size.

**Table 4:** Actual and predicted values of *k* and *m* at varying times and solid concentrations, along with corresponding d_50_, d_80_, and d_90_ values.

Grinding time	Solid, %	Actual		Predicted		Actual			predicted		
		k	m	k	m	D_50_	D_80_	D_90_	D_50_	D_80_	D_90_
1	20	0.1529	4.6867	0.1469	4.6918	5.06	11.06	15.59	5.31	11.54	16.26
2.75	20	0.2787	4.7841	0.2737	4.7817	3.13	6.42	8.90	3.18	6.52	9.06
4.5	20	0.3672	4.8263	0.3794	4.8244	2.49	4.98	6.87	2.40	4.82	6.65
7.5	20	0.5129	4.7882	0.5118	4.7875	1.71	3.49	4.85	1.71	3.50	4.86
10	20	1.536	5.6095	1.4400	5.5525	1.11	1.70	2.15	1.14	1.77	2.26
14	20	8.9064	11.602	8.6863	11.6200	0.86	0.96	1.04	0.89	0.99	1.07
1	33.3	0.1803	4.7188	0.1882	4.7115	4.47	9.56	13.40	4.25	9.12	12.80
2.75	33.3	0.3055	4.8126	0.3012	4.8154	2.95	5.95	8.22	2.99	6.04	8.34
4.5	33.3	0.3876	4.8696	0.3930	4.8721	2.47	4.83	6.62	2.44	4.77	6.54
7.5	33.3	0.5104	4.8568	0.5015	4.8593	1.85	3.65	5.00	1.88	3.71	5.10
10	33.3	1.5937	5.4437	1.5674	5.4737	0.96	1.54	1.97	0.99	1.58	2.02
14	33.3	5.6844	9.12	5.6811	8.9197	0.92	1.07	1.20	0.88	1.04	1.16
17	33.3	8.7515	11.491	8.7874	11.5040	0.86	0.97	1.05	0.86	0.97	1.05
1	50	0.1475	4.6455	0.1490	4.6481	4.97	11.18	15.88	4.94	11.09	15.74
2.75	50	0.2483	4.7696	0.2445	4.7696	3.45	7.144	9.94	3.50	7.25	10.09
4.5	50	0.329	4.8442	0.3189	4.8439	2.83	5.62	7.73	2.92	5.79	7.97
7.5	50	0.3886	4.8624	0.3975	4.8612	2.44	4.80	6.59	2.38	4.69	6.44
10	50	0.5072	4.9228	0.4670	4.8537	1.99	3.79	5.17	2.02	3.98	5.46
14	50	0.5659	4.8991	0.6631	5.0081	1.74	3.36	4.59	1.65	3.03	4.08
17	50	0.7921	5.1083	0.8312	5.1237	5.06	11.06	15.59	5.31	11.54	16.26
22	50	1.3043	5.4035	1.1514	5.3158	3.13	6.42	8.90	3.17	6.52	9.06
27	50	1.4587	5.4696	1.5217	5.5073	2.48	4.98	6.87	2.40	4.82	6.65

**Table 5:** Comparison between operational parameters of the present work and those reported in other studies, highlighting particle size and energy consumption.

Mill type, specification	Ore	feed size, µm	product size, µm	Energy, kWh/t	Grinding condition	Ref.
Laboratory stirred mill Union Process, 9.5 L	Copper	-500 + 75 μm	**~ 1 μm**	1225	Solid concentration of 33.3%, Stirrer of 500 rpm (4.71 m/s), grinding time 17 h.	Present work
NETZSCH laboratory vertical stirred mill, 5.3 L	Chromite	-150 + 106 μm	11.6 μm	21.8	Solid concentration 50.1%, Stirrer speed 622 rpm	^2^
Laboratory-scale stirred media Union Process, Akron, Ohio, 9.5 L	Coal	-24.4 μm	P_80_= 5.9 μm	309	Solid concentration of 30%, Stirrer of 340 rpm, grinding time 64 min.	^14^
	Coal	-23.40 μm	10.29 μm	397	Solid concentration of 30%, Stirrer of 340 rpm, grinding time 52 min.	^53^
crew type batch vertical stirred mill (JKMRC), A steel pot (380 mm diameter)	Limestone	-580 μm	Less than 100 μm	10.8	Higher mill tip speed of 3 m/s, lower solids concentration of 50% generates finer particle size	^27^
lab-scale grinding mill (IMERYS), 2.9 L	Calcium carbonate	D_80_ = 62.8 μm	Approx. 1.8 μm	300	Solid concentration of 65%, Stirrer tip speed of 5.23 m/s	^28^
Union Process STD-01 batch-type laboratory scale pin-type vertical stirred mill with, 750 cc	Calcite	D_50_ = 5.4 μm	70% < 1 μm	1340	Solid concentration of 25%, Stirrer speed of 600 rpm, 2 h. grinding time	^55^
	Calcite	D_50_= 10 μm	P_50_= 3.96 μm	34.5	Stirrer speed of 573 rpm, grinding time of 11.18, Media filling ratio of 63, and Solid mass fraction of 11.52%	^29^
Batch vertical laboratory scale stirred mill	refractory Au/Ag	d80 = 60 μm	P_80_= 3.37 μm		Stirrer speed of 745 rpm, ball charge ratio of 80%, grinding time of 10.5 min	^30^
vertical stirred mill (KMD-1B, manufactured by Korea	Calcite powder	D_50_ = 10.82 μm	Down to 350 nm		Grinding time 480 min, rpm 700, 15% Solid	^31^
Laboratory scale stirred mill	Bauxite ore	-780 μm	P_50_ = 5 μm	~ 174	At a stirrer speed of 1500 rpm	^54^
Laboratory stirred mill (Hosokawa Alpine 90), 1.12 L	Titanium dioxide	-1 μm	0.3 μm	100	Impeller tip speed of 9.2 m/s, and Solid concentration of 52%	^56^

## Conclusion

This study investigates the effects of key operational parameters—grinding time, stirrer tip speed, solid concentration, and feed size—on grinding efficiency, assessed through specific energy inputs, in the stirred milling of An Egyptian copper ore. The particle size distribution (PSD) of the ground products was modeled using the Gates–Gaudin-Schuhmann (GGS) model and the Rosin-Rammler-Benne (RRB) function. The results are summarized as follows:

1. Increasing impeller tip speed in stirred milling significantly enhances grinding performance by reducing both particle size and specific energy consumption. Within the range of 2.54 to 4.71 m/s, higher impeller speeds notably decrease processing time and energy usage, with the most substantial improvements observed between 2.54 m/s and 3.62 m/s. At an energy input of 100 kWh/t, the particle size decreases from 8.4 μm to 6.5 μm, and the processing time is reduced from 9 h to 2 h and 45 min. These results underscore the benefits of optimizing impeller speed for more efficient grinding operations.
2. Optimizing solid concentration is essential for enhancing grinding efficiency and achieving ultrafine particles in a stirred mill. For the ore tested in this study, the relationship between specific energy (kWh/t) and particle size was demonstrated, with the finest particles (~ 1 μm) obtained at a 33.3% solids concentration after 17 h of grinding, consuming approximately 1225 kWh/t. Beyond this point, no significant reduction in particle size was observed.
3. Stress intensity increases significantly with higher stirrer speeds, rising from 9 × 10⁻⁴ Nm at a tip speed of 2.54 m/s to 31.13 × 10⁻⁴ Nm at 4.71 m/s. Similarly, stress intensity intensifies as the solid concentration in the slurry decreases, increasing from 31.13 × 10⁻⁴ Nm at 50% solids to 35.92 × 10⁻⁴ Nm at 20% solids.
4. Coarser feed particles (− 500 + 75 μm) are easier to grind than finer particles (− 75 μm), likely due to differences in ore characteristics and reduced viscosity buildup. Achieving a particle size of P_80_ ~ 1 μm, with a reduction ratio of 410, required approximately 1025 kWh/t for coarser feed, while finer feed, with a reduction ratio of 49, required 1500 kWh/t.
5. For the ore tested in this study, under the optimal conditions of a high impeller tip speed of 4.71 m/s and 33.3% solid concentration, the GGS model is not suitable for describing the particle size distributions (PSDs) of the product at any of the tested grinding times. In contrast, the RRB model provides a good fit for grinding to a product size of d_100_ > 6 μm (d_50_ > 1.4 μm). However, it does not accurately model particle sizes below 6 μm.
6. The experimental data demonstrated a linear relationship between the natural logarithm of the cumulative retained fraction and particle size (µm), and the proposed model effectively describes all product particle size distributions (PSDs) across varying solid concentrations and grinding durations. This model, expressed by the equation $\:R={exp}^{-kX+m}$, provides an accurate prediction of PSDs, including particle sizes down to 1 micron, and is supported by statistical equations that predict the values of k and m based on grinding time and solid concentration. Future studies are encouraged to incorporate statistical methods to deepen the understanding of interactions between parameters. Additionally, expanding the model to account for rotational speed and other critical factors is recommended to enhance its applicability.

## Data Availability

The datasets generated and/or analysed during the current study are not publicly available due to institutional roles and confidential conditions but are available from the corresponding author on reasonable request.
